# Deep brain stimulation in disorders of consciousness: 10 years of a single center experience

**DOI:** 10.1038/s41598-023-46300-y

**Published:** 2023-11-09

**Authors:** Darko Chudy, Vedran Deletis, Veronika Paradžik, Ivan Dubroja, Petar Marčinković, Darko Orešković, Hana Chudy, Marina Raguž

**Affiliations:** 1 Department of Neurosurgery, Dubrava University Hospital, Zagreb, Croatia; 2 Department of Surgery, School of Medicine, University of Zagreb, Zagreb, Croatia; 3 Albert Einstein College of Medicine, New York, USA; 4 Brain Trauma Unit, Specialty Hospital for Medical Rehabilitation, Krapinske Toplice, Croatia; 5 Department of Neurology, Dubrava University Hospital, Zagreb, Croatia; 6 School of Medicine, Catholic University of Croatia, Zagreb, Croatia

**Keywords:** Disorders of consciousness, Hypoxic-ischaemic encephalopathy

## Abstract

Disorders of consciousness (DoC), namely unresponsive wakefulness syndrome (UWS) and minimally conscious state (MCS), represent severe conditions with significant consequences for patients and their families. Several studies have reported the regaining of consciousness in such patients using deep brain stimulation (DBS) of subcortical structures or brainstem nuclei. Our study aims to present the 10 years’ experience of a single center using DBS as a therapy on a cohort of patients with DoC. Eighty Three consecutive patients were evaluated between 2011 and 2022; entry criteria consisted of neurophysiological and neurological evaluations and neuroimaging examinations. Out of 83, 36 patients were considered candidates for DBS implantation, and 32 patients were implanted: 27 patients had UWS, and five had MCS. The stimulation target was the centromedian-parafascicular complex in the left hemisphere in hypoxic brain lesion or the one better preserved in patients with traumatic brain injury. The level of consciousness was improved in seven patients. Three out of five MCS patients emerged to full awareness, with the ability to interact and communicate. Two of them can live largely independently. Four out of 27 UWS patients showed consciousness improvement with two patients emerging to full awareness, and the other two reaching MCS. In patients with DoC lasting longer than 12 months following traumatic brain injury or 6 months following anoxic-ischemic brain lesion, spontaneous recovery is rare. Thus, DBS of certain thalamic nuclei could be recommended as a treatment option for patients who meet neurological, neurophysiological and neuroimaging criteria, especially in earlier phases, before occurrence of irreversible musculoskeletal changes. Furthermore, we emphasize the importance of cooperation between centers worldwide in studies on the potentials of DBS in treating patients with DoC.

## Introduction

Improved logistic factors in emergency medicine to treat life-threatening conditions such as neurotrauma, cardiac arrest, and ischemic brain lesions increases survivors. However, the outcome in some patients varies, from behavioral or cognitive disturbances to severe neurological deficits or disorders of consciousness^1^.

Disorder of consciousness (DoC) is a term used for patients who are not awake or have difficulty maintaining wakefulness and have altered or impaired awareness of themselves and their surroundings. DoC can occur due to several incidents, such as traumatic brain injury, global ischemic brain lesion, cerebrovascular insult, or non-traumatic intracranial bleeding^2–4^. Prognosis depends on the cause and the extent of brain damage, where the global ischemic lesion has a substantially less favorable prognosis than traumatic brain injury^3,5,6^. Furthermore, the categorization of DoC depends not only on neurological status but also on how much time has passed since the incident^2–4^. DoC is classified as unresponsive wakefulness syndrome (UWS), previously known as an apallic syndrome, coma vigile, and vegetative state is characterized by arousal without awareness. A minimally conscious state (MCS) is defined as a reproducible but inconsistent awareness^5,7,8^.

Moreover, the MCS has been additionally classified according to patients' ability to verbalize and communicate in the MCS minus (MCS−) and MCS plus (MCS+) patients intelligibly or intentionally^9^.

Nowadays, a number of scales are being used to assess the severity of DoC^10^. The instruments for assessment of DoC must be precise enough to rate even a slight improvement of conscious state and practical enough that the time duration to evaluate is optimal. It is essential to emphasize the proper and adequate scale selection and meticulous usage of the selected scale because of the possibility of an unexpectedly high proportion of misdiagnosis in differentiating MCS ad UWS, as previously described^11,12^. The most commonly used scales are the Coma Recovery Scale-Revised (CRS-R), an internationally accepted scale for assessing consciousness in patients with severe brain injury^13^ and the Coma/Near Coma (C/NC) Scale^14^, both used in our study to enable our results to be comparable with the results of other centers that also treated patients with DoC.

Effective treatment for patients with DoC is still ambiguous, and many methods, including deep brain stimulation (DBS), are in the experimental phase. The therapies for patients with DoC are quite limited, especially evidence-based ones. Therefore, physical therapy is still the cornerstone of medical care for patients with DoC.

Usage of chronic electrical stimulation for patients with DoC began in the 1960s with a case report published by Hassler and al. 1969, followed by McLardy in 1969 and Sturm in 1979^15–17^. Although arousal effects accompanied the neurostimulation, few clinical signs of improvement were reported. Cohadon described 25 patients with DoC in a study published in 1993^18^. Additionally, in 1993, Hosobuchi and Yingling described some improvements in one patient without restoring consistent communication^19^. Tsubokawa and Yamamoto published several papers between 1990 and 2013, reporting their results of DBS of the centromedian-perifascicular (CM-pf) nuclei of the thalamus in 24 patients and DBS of the mesencephalic reticular formation in two patients^20–23^. After these heterogeneous results of early DBS studies, Schiff et al. proposed to perform DBS in patients with MCS instead of UWS, since MCS patients might have more intact functional brain networks and therefore, a larger capacity for functional recovery^24^. A case report of an MCS patient six years after a traumatic brain injury with evidence of behavioral improvement using DBS was published in 2007 by Schiff et al.^25^. In 2016 a group of authors from Pavia presented their results of DBS in three patients with DoC, with an improvement of CSR-R score reported in two UWS and one MCS patient^26^. In 2018 we published our results of 14 patients with DoC using DBS of CM-pf nucleus, where three of four MCS and one UWS patient reached the level of awareness^27,28^. Lastly, an interesting paper from the Amsterdam group targeting CM-pf nuclei of thalamus bilaterally in one MCS patient, resulting in a direct increase in arousal^29^. It is important to emphasize that these studies were performed in heterogeneous groups of patients with different etiologies, often without standardized assessment scales of consciousness, and with various follow-up times. Therefore, it is difficult to interpret and generalize their findings.

We started our study at the beginning of 2011. The aim was to try to establish prognostic factors and improve the inclusion criteria for patients with DoC who are candidates for DBS. Furthermore, we aimed to find the best timing to start with DBS after an initial incident in patients who developed irreversible changes to their locomotor system in a very short time. In this study, we present the long-term results of the cohort of patients with DoC treated with DBS of the CM-pf nuclei in the Department of Neurosurgery, Dubrava, University Hospital, Zagreb, Croatia, from 2011 to 2022 focusing on clinical improvement.

## Patients and methods

**Figure 1:**
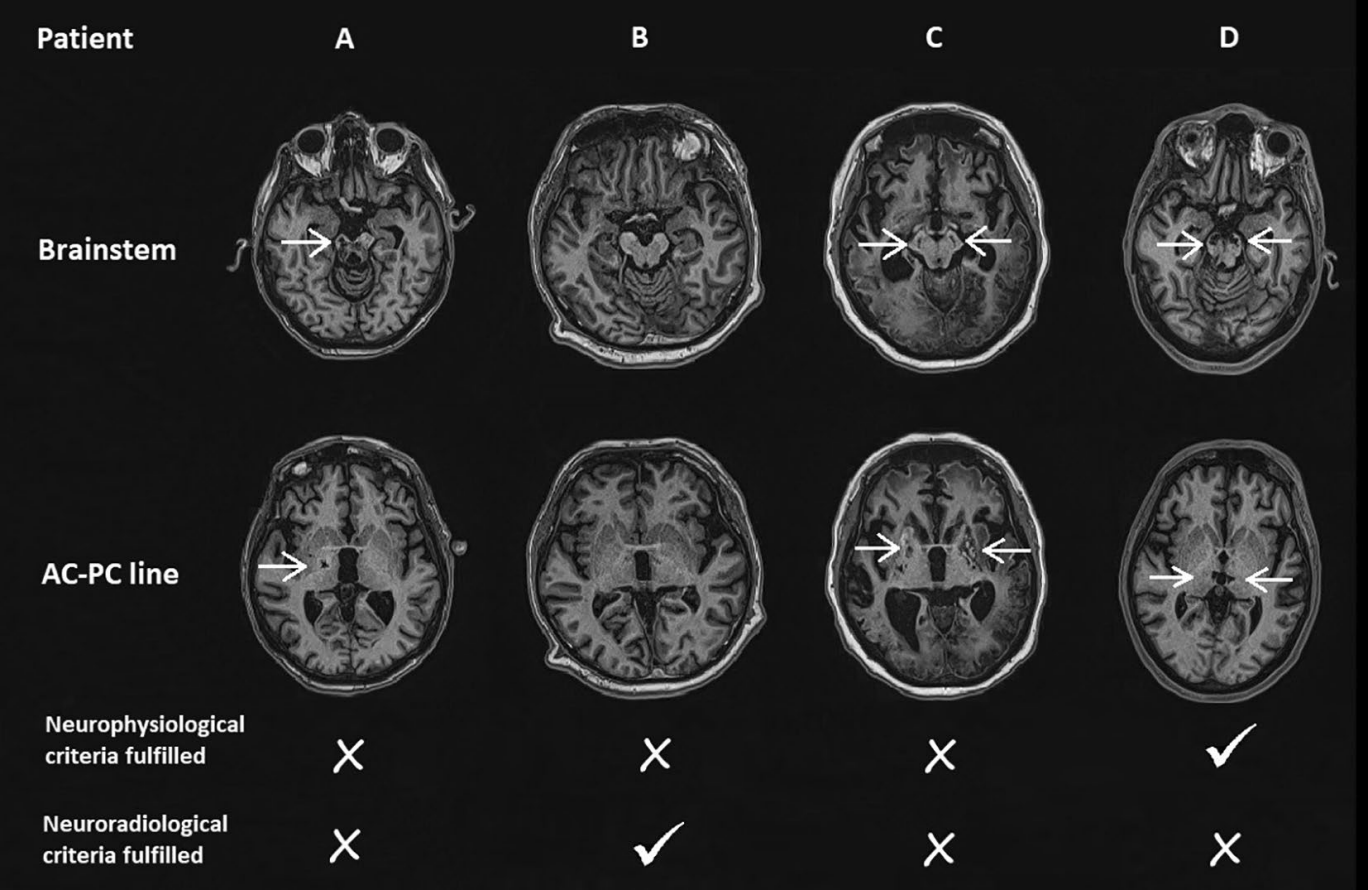
Brain MRIs of patients who weren’t candidates for DBS implantation, T1 MPRAGE, axial plane. (**A**) Female patient, 51 yrs, DoC due to intracerebral hemorrhage; significant lesion observed at the level of brainstem and thalamus. Patient didn’t fulfil both neurophysiological and neuroradiological criteria. (**B**) Male patient, 57 yrs, DoC due to cardiac arrest, no significant lesion on MRI. Patient didn’t fulfil neurophysiological criteria. (**C**) Female patient, 46 yrs, DoC due to cardiac arrest; global ischemic cortical, basal ganglia and brainstem changes. Patient didn’t fulfil both neurophysiological and neuroradiological criteria. (**D**) Male patient, 36 yrs, DoC due to septic embolization of the brain; significant lesion observed at the level of brainstem and thalamus. Patient fulfil neurophysiological criteria. White arrows represents lesions visible on different levels of brain MRI.

Patients selection was made based on multiple tests as follows: (1) neurophysiologic evaluations that consisted of somatosensory evoked potentials (SEPs), motor evoked potentials (MEPs), brainstem auditory evoked potentials (BAEPs), and 12/24 h EEG; (2) clinical evaluation, consisting of evaluation using the C/NC and CRS-R scale; and (3) neuroimaging by magnetic resonance imaging (MRI). The inclusion criteria for patients were meeting the diagnostic criteria for DOC^30^; the patients who were inconsistently or partially responsive to simple commands were classified as MCS, while patients who consistently weren’t able to respond to any simple command were classified as UWS. Furthermore, patients were in hemodynamic and respiratory stable condition, with DOC duration for minimally 6 weeks, with the absence of lesions i.e. hemorrhages or infarction in the brainstem, diencephalon, or basal ganglia, visible on MRI (Fig. 1). Specific neurophysiologic entry criterion included preserved cortical SEPs and MEPs at least from the upper extremities, even with prolonged latency and low amplitude, and BAEPs whose values were not within the normal limits. In addition, EEG demonstrated periods of beta activity i.e. desynchronization.

In our previous publication, we presented the clinical evaluation according to the C/NC scale, while from 2018 we implemented the CRS-R scale; all included patients were scored using the mentioned scales as well as based on extensive medical history and a number of videos.

Eighty Three consecutive patients were screened according to the above-mentioned criteria and 36 patients were determined as candidates, while 47 patients were determined as non-candidates for CM-pf DBS implantation. Clinical data of both groups are presented in Table 1. Four patients were excluded in the group that fulfilled neurophysiologic, clinical, and imaging criteria in because they died during the Covid-19 pandemic, waiting for DBS, mainly due to extensive cachexia and lacking adequate nursing and physical therapy. Thus, CM-pf DBS was implanted in 32 patients. Demographic and clinical characteristics of the patients who fulfilled neurophysiologic, clinical, and imaging criteria and underwent CM-pf DBS are presented in Table 2.

**Table 1:** Summary of demographic and clinical characteristics of all patients included in the study.

	Candidates	Non-candidates
No.	36	47
Range of age	7–81	12–64
Male/Female (n)	21/15	26/21
Traumatic/Anoxic (n)	10/26	15/32
MCS/UWS	5/31	0/47

**Table 2:** Summary of demographic and clinical characteristics of the patients who fulfilled neurophysiologic, clinical, and imaging criteria and underwent CM-pf DBS.

	Unresponsive wakefulness syndrome (UWS)	Minimally consciousness state (MCS)	Overall
Patients (n)	27	5	32
Age at injury (year)	38.74 ± 17.65 (range 12–66)	19.20 ± 4.02 (range 15–24)	35.69 ± 17.74 (range 12–66)
Male/female (n)	18/9	4/1	22/10
Traumatic/anoxic (n)	8/19	1/4	9/23
DBS after injury (months)	9.81 ± 9.79 (range 2–48)	31.20 ± 59.26 (range 2–137)	13.16 ± 24.41 (range 2–137)

**Figure 2:**
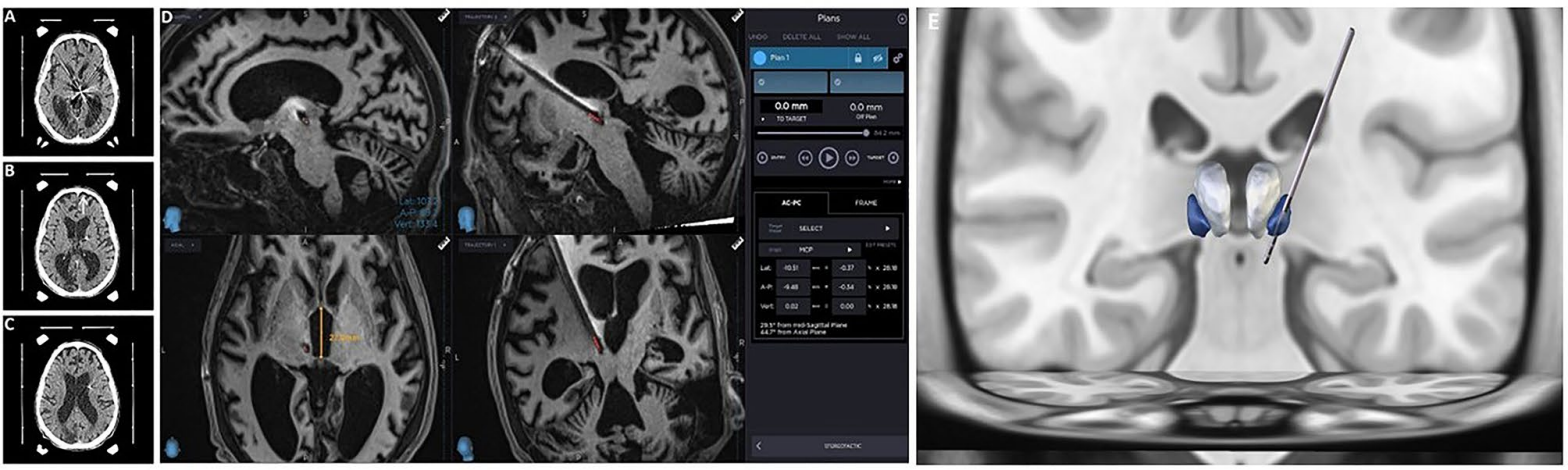
Position of the implanted lead in CM-pf, presented in axial plain on immediate postoperative framebased CT on the level of the thalamus (**A**), at the level of lateral ventricles with white arrow pointing to intracranial air and absence of brain shift (**B**), and at the level of lateral ventricles (**C**). Position of the implanted lead in CM-pf in transversal and sagittal plane presented on early postoperative MRI using Stealth Station (Medtronic), with coordinates on the right side (**D**). Additionally, reconstructed lead and its position regarding CM (blue) and MD-pf (light grey) nuclei of the thalamus using Lead DBS software (**E**).

The preoperative MRI revealed signs of hypoxic-ischemic encephalopathy with marked brain atrophy in all 32 patients. In nine patients with significant posttraumatic lesions, the DBS lead was implanted in the more preserved hemisphere, otherwise in the dominant hemisphere, usually left. Due to our protocol, immediate postoperative frame-based CT is obtained after CM-pf implantation serving as a confirmation tool for target and trajectory, but more importantly, to verify absence of postoperative hemorrhage and minimal or no pneumocephalus, therefore brain shift^27,28^ (Fig. 2.) Surgical targeting and CM-pf implantation procedure were described previously; the target coordinates were calculated on immediate preoperative CT scan with Leksell frame, as follows: 4.5 mm anterior to posterior commissure, 1 mm below the intercommissural line and 4 mm lateral to the ventricular wall, according to Schaltenbrand-Bailey stereotactic atlas; in this way, we encounter the problem of widened third ventricle due to brain atrophy^27,28^. The lead position was confirmed using postoperative brain MRI acquired at most one month after the implantation. For lead reconstruction and positioning, we used Lead DBS software and THalamus Optimized Multi Atlas Segmentation (THOMAS) atlas, although the mentioned software is not intended for any clinical or diagnostic use (Fig. 2)^31,32^.

In our cohort, we implanted either Medtronic lead models 3387 and 3389, or Boston Scientific Vercise directional leads, and a non-rechargeable pectoral pulse stimulator. Monopolar stimulation was started on the third postoperative day. Patients 1–14 were implanted with Medtronic lead models 3387, while patients 15–26 were implanted with Medtronic lead models 3389. Using a stimulation frequency of 25–40 Hz and a pulse width of 120–210 μs, we tested all 4 contacts with voltage varying from 2.5 to 3.5 V up to 5 V, in order to elicit the strongest arousal reaction. In all patients with Medtronic leads, single contact was chosen (in most cases 0 or 1). Patients 27–32 had DBS implantation with Boston Scientific Vercise directional leads, and a similar approach was used to elicit the strongest arousal reaction (frequency 25–40 Hz, pulse width of 120–210 μs, and current strength 3.0–5.0 mA). Arousal reaction consists of eye-opening (if the patient’s eyes were closed), with mydriasis and different facial expressions compared with before stimulation. Some of the patients turned their heads in one direction and had an elevation of their blood pressure along with heart rate elevation^27,28^. Furthermore, if an arousal effect could not be elicited using single contact, more contacts were activated, covering a greater volume of the thalamus (i.e. more thalamic nuclei) until an arousal reaction was obtained. Stimulation was applied for 30 min every 2 h during the daytime. During the night, the stimulation was completely stopped with the aim of creating cycles of sleep and awake, like previously described^20^.

Patients’ follow-up was done using clinical evaluations at one, three, and six months after the surgery and then every year. Patients who were not implanted because they did not fulfill neurophysiologic, clinical, and imaging criteria were followed up at least one year after the initial admission.

This study was carried out in accordance with the recommendations of the ethics board of the Dubrava University Hospital and School of Medicine the University of Zagreb. Written informed consent was obtained for all patients by their caregivers in accordance with the Declaration of Helsinki and after a detailed discussion about the risk and expectations of this DBS procedure which is still in an experimental phase. The protocol was approved by the Institutional Review Board of the Dubrava University Hospital, Zagreb, Croatia (2409-20) and the Institutional Review Board of the Dubrava University Hospital, Zagreb, Croatia (2002-01).

Data analysis was performed using the MedCalc Statistical Software version 12.5.0 (MedCalc Software, Ostend, Belgium; https://www.medcalc.org). The Kolmogorov–Smirnov test assessed distribution. Student t-test was used to present the difference between the two groups, while one-way ANOVA was used to compare prospective data. The statistical significance was set at *p* < *0.05*.

## Results

**Figure 3:**
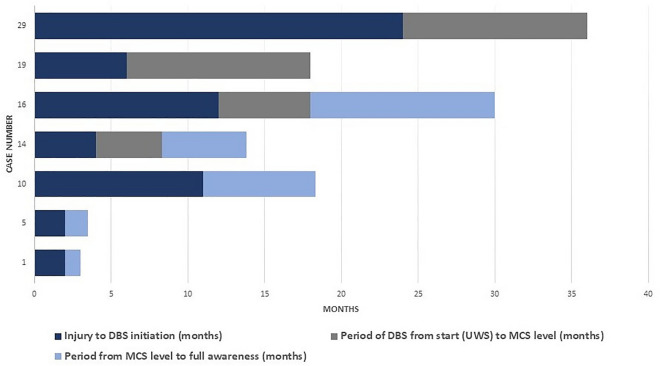
Time course of recovery in both UWS and MCS patients, after CM-pf DBS, presented in months.

All 32 consecutive patients who fulfilled neurophysiologic, clinical, and imaging criteria and were implanted with unilateral CM-pf DBS; in 29 patient DBS was implanted in the left hemisphere, in two patients in the right hemisphere, and in 1 patient we implanted bilaterally. DBS elicited an arousal reaction during stimulation in each patient, as described previously^27,28^. In two UWS patients and one MCS we observed adverse events like seizures which were controlled with antiepileptic drugs, allowing DBS to proceed. In recovered patients, the arousal reaction gradually disappeared as their level of consciousness increased. The recovery of four patients was described previously^27,28^. Three MCS patients emerged to full awareness, with the ability to interact and communicate, while two of them can live largely independently. Patient No 1 is now completely recovered, as well as Patient No 5. Patient No 10 suffered a traumatic brain injury and is still severely motor disabled and needs assistance in her activities of daily living. Four UWS patients showed consciousness improvement. Two patients emerged to full awareness; patients No 15 and No 16 are still bedridden and need assistance in activities of daily living. Two UWS patients reached MCS level, No 19 and 29. In Table 3 we summarized improved patients while their improvement dynamic was schematically presented in Fig. 3. Age of injury in patients who recovered was 22.86 ± 15.87, with average time to DBS 8.71 ± 7.85 (Table 3). In the presented cohort, the shortest follow-up was 4 months, as the patient has died, while the longest follow-up was over ten years (Tables 3, 4).The individual results of all 32 patients were shown in Table 4. For better clarity we divided Table 4 into patients with DOC after cardiac arrest and traumatic brain injuries. Additionally, we divided patient into acute DOC and chronic of more than 6 months for CA and more than 12 months for TBI.

**Table 3:** Summary of data for patients who improved after CM-pf DBS.

Case no.	Gender	Cause of injury	Age at injury (yrs)	Time to DBS (months)	RDR before DBS	CRS-R before DBS	RDR 1 year after DBS	CRS-R 1 year after DBS	Follow-up (months)
1	M	CA	17	2	2.0/1 MCS -	7	0 aware	23	120
5	M	CA	23	2	1.8/1 MCS -	11	0 aware	23	108
10	F	TBI	15	11	1.6/1 MCS -	8	0 aware	21	96
14	F	CA	16	4	2.6/2 UWS	3	0 aware	21	96
16	F	TBI	19	12	2.6/2 UWS	6	0 aware	21	52
19	M	CA	12	6	2.4/2 UWS	5	1.2/1 MCS+	18	36
29	M	CA	58	24	2.2/2 UWS	7	1.2/1 MCS+	14	16

*M* male, *F* female, *CA* cardiac arrest, *TBI* traumatic brain injury, *UWS* unresponsive wakefulness syndrome, *MCS* minimally conscious state, *RDR* Rappaport disability rate, *CRS-R* Coma Recovery Scale-Revised.

**Table 4:** Overall patients included in study, divided in four groups: cardiac arrest (CA) patients with acute DoC, CA patients with chronic DoC, traumatic brain injury (TBI) patients with acute DoC and TBI patients with chronic DoC.

Cause of injury, acute/chronic DoC	Case No	Gender	Age at injury (years)	Time to DBS (months)	Implantation side	RDR before DBS	CRS-R before DBS	RDR 1 year after DBS	CRS-R 1 year after DBS	Time of follow-up (months)
CA patients with acute DOC (< 6 months)	1*	M	17	2	L	2.0/1 MCS−	7	0 aware	23	120
	3*	F	49	6	L	3.6/4 UWS	4	2.6/2 UWS	7	114
	4*	M	20	3	R + L	3.4/3 UWS	4	2.0/3 UWS	7	109
	5*	M	23	2	L	1.8/1 MCS−	11	0 aware	23	108
	6*	M	59	2	L	3.8/4 UWS	4	3.0/3 UWS	7	56
	13*	M	17	3	L	3.4/3 UWS	4	2.2/2 UWS	7	96
	14*	F	16	4	L	2.6/2 UWS	3	0 aware	21	96
	15	M	24	4	L	1.0/1 MCS−	13	1.0/1 MCS -	14	65
	17	M	33	6	L	2.6/2 UWS	5	2.0/2 UWS	7	65
	18	M	53	3	L	3.2/3 UWS	4	2.4/2 UWS	6	65
	19	M	12	6	L	2.4/2 UWS	5	1.2/1 MCS+	18	36
	25	M	66	2	L	3.4/3 UWS	5	3.0/3 UWS	8	40
	27	M	62	2	L	3.0/3 UWS	4	2.4/2 UWS	7	15, died
	28	M	16	2	L	3.0/3 UWS	4	2.2/2 UWS	6	13
	31	F	55	6	L	3.4/3 UWS	4	2.6/2 UWS	6	13
CA patients with acute DOC (> 12 months)	7*	M	34	7	L	3.0/3 UWS	4	2.6/3 UWS	6	18, died
	9*	F	39	7	L	3.8/4 UWS	4	Died	Died	4, died
	30	M	64	12	L	3.4/3 UWS	4	2.6/2 UWS	5	14
	11*	M	17	137	L	1.0/1 MCS−	17	1.0/1 MCS+	17	96
	21	M	14	13	L	2.6/2 UWS	4	2.2/2 UWS	6	96
	23	F	52	16	L	3.6/4 UWS	3	2.2/2 UWS	6	20
	24	F	40	48	L	3.6/4 UWS	3	3.0/3 UWS	4	22
	29	M	58	24	L	2.2/2 UWS	7	1.2/1 MCS+	14	16
TBI patients with acute DoC (< 12 months)	2*	M	25	5	L	3.6/4 UWS	6	3.4/3 UWS	8	116
	10*	F	15	11	L	1.6/1 MCS−	8	0 aware	21	96
	16	F	19	12	R	2.6/2 UWS	6	0 aware	21	65
	22	M	47	4	L	3.2/3 UWS	6	1.4/1 UWS	12	12, died
	32	M	57	6	L	3.4/3 UWS	4	3.0/3 UWS	6	13
TBI patients with acute DoC (> 12 months)	8*	F	28	17	L	2.2/2 UWS	6	2.1/2 UWS	8	53
	12*	M	43	21	L	3.2/3 UWS	5	3.0/3 UWS	6	33, died
	20	M	23	15	L	3.4/3 UWS	4	3.0/3 UWS	6	31
	26	F	45	13	R	3.8/4 UWS	4	3.2/3 UWS	6	60, died

Chronic DoC: > 6 months for CA and > 12 months for TBI. *Primary cohort, Chudy et al. ^27^.

*M* male, *F* female, *CA* cardiac arrest, *TBI* traumatic brain injury, *UWS* unresponsive wakefulness syndrome, *MCS* minimally conscious state, *RDR* Rappaport disability rate, *CRS-R* Coma Recovery Scale-Revised.

As mentioned, the patients were clinically evaluated using C/NC, and CRS-R scale one, three, and six months postoperatively, as well as after the first, second, and third year (Table 5). C/NC scale subcategories included auditory, command responsivity, visual, threat, olfactory, tactile, pain and vocalization, while CRS-R scale subcategories included auditory, visual, motor, oromotor, communication and arousal functions.

**Table 5:** Clinical evaluation of all patients included in study, one, three, and six months after surgery and the first, second, and third year postoperatively.

Case no.	RDR before DBS	CRS-R before DBS	RDR one month post DBS	CSR-R one month post DBS	RDR three months post DBS	CSR-R three months post DBS	RDR six months post DBS	CSR-R six months post DBS	RDR one year post DBS	CSR-R one year post DBS	RDR two years post DBS	CSR-R two years post DBS	RDR three years post DBS	CSR-R three years post DBS
1	2.0/1	7	0	23	0	23	0*	23*	0	23	0	23	0	23
2	3.6/4	6	3.6/4	6	3.6/4	6	3.6/4	6	3.4/3	8	3.2/3	8	3.2/3	8
3	3.6/4	4	3.0/3	6	2.8/2	7	2.6/2	7	2.6/2	7	2.6/2	7	2.6/2	7
4	3.4/3	4	3.0/3	5	2.8/3	6	2.0/3	7	2.0/3	7	2.0/3	7	2.0/3	7
5	1.8/1	11	0	23	0	23	0*	23*	0	23	0	23	0	23
6	3.8/4	4	3.6/4	5	3.2/3	6	3.0/3	7	3.0/3	7	3.0/3	7	3.0/3	7
7	3.0/3	4	2.8/3	5	2.8/3	5	2.6/3	6	2.6/3	6	Died	Died	Died	Died
8	2.2/2	6	2.2/2	7	2.1/2	7	2.1/2	8	2.1/2	8	2.1/2	8	2.1/2	8
9	3.8/4	4	3.6/4	5	3.6/4	5	Died	Died	Died	Died	Died	Died	Died	Died
10	1.6/1	8	1.4/1	9	1.2/1	11	1.0/1	16	0	21	0*	21*	0	21
11	1.0/1	17	1.0/1	17	1.0/1	17	1.0/1	17	1.0/1	17	1.0/1	17	1.0/1	17
12	3.2/3	5	3.2/3	5	3.0/3	6	3.0/3	6	3.0/3	6	3.0/3	6	Died	Died
13	3.4/3	4	3.0/3	5	2.6/2	6	2.2/2	7	2.2/2	7	2.2/2	7	2.2/2	7
14	2.6/2	3	2.4/2	4	2.2/2	6	1.0/1	16	0	21	0*	21*	0	21
15	1.0/1	13	1.0/1	13	1.0/1	13	1.0/1	13	1.0/1	14	1.0/1	14	1.0/1	14
16	2.6/2	6	2.6/2	6	2.4/2	7	1.0/1	16	0	21	0*	21*	0	21
17	2.6/2	5	2.6/2	5	2.4/2	6	2.2/2	6	2.0/2	7	2.0/2	7	2.0/2	7
18	3.2/3	4	3.0/3	4	2.8/2	5	2.6/2	6	2.4/2	6	2.4/2	6	2.4/2	6
19	2.4/2	5	2.4/2	5	2.0/2	6	1.6/1	13	1.2/1	18	1.0	18	1.0*	18*
20	3.4/3	4	3.4/3	4	3.4/3	4	3.2/3	5	3.0/3	6	3.0/3	6	3.0/3	6
21	2.6/2	4	2.4/2	5	2.4/2	5	2.2/2	6	2.2/2	6	2.2/2	6	2.2/2	6
22	3.2/3	6	3.0/3	7	2.2/2	8	2.0/2	9	1.4/1	12	Died	Died	Died	Died
23	3.6/4	3	3.0/3	4	2.8/2	5	2.4/2	6	2.2/2	6	2.2/2	6	2.2/2	6
24	3.6/4	3	3.4/3	3	3.2/3	4	3.0/3	4	3.0/3	4	3.0/3	4	3.0/3	4
25	3.4/3	5	3.2/3	6	3.2/3	6	3.0/3	7	3.0/3	8	3.0/3	8	3.0/3	8
26	3.8/4	4	3.8/4	4	3.6/3	5	3.4/3	5	3.2/3	6	3.2/3	6	3.2/3	6
27	3.0/3	4	2.8/2	5	2.6/2	6	2.6/2	6	2.4/2	7	Died	Died	Died	Died
28	3.0/3	4	2.8/2	5	2.6/2	5	2.4/2	6	2.2/2	6				
29	2.2/2	7	2.0/2	7	2.0/2	7	1.6/1	11	1.2/1	14				
30	3.4/3	4	3.0/3	4	2.8/2	5	2.6/2	5	2.6/2	5				
31	3.4/3	4	3.0/3	5	2.6/2	6	2.6/2	6	2.6/2	6				
32	3.4/3	4	3.2/3	5	3.0/3	6	3.0/3	6	3.0/3	6				

In patients 28–32 follow-up is still ongoing, and is shorter than 2 years. Asterisk* markers when the use of the daytime DBS was stopped for the responders.

*RDR* Rappaport disability rate, *CRS-R* Coma Recovery Scale-Revised.

**Figure 4:**
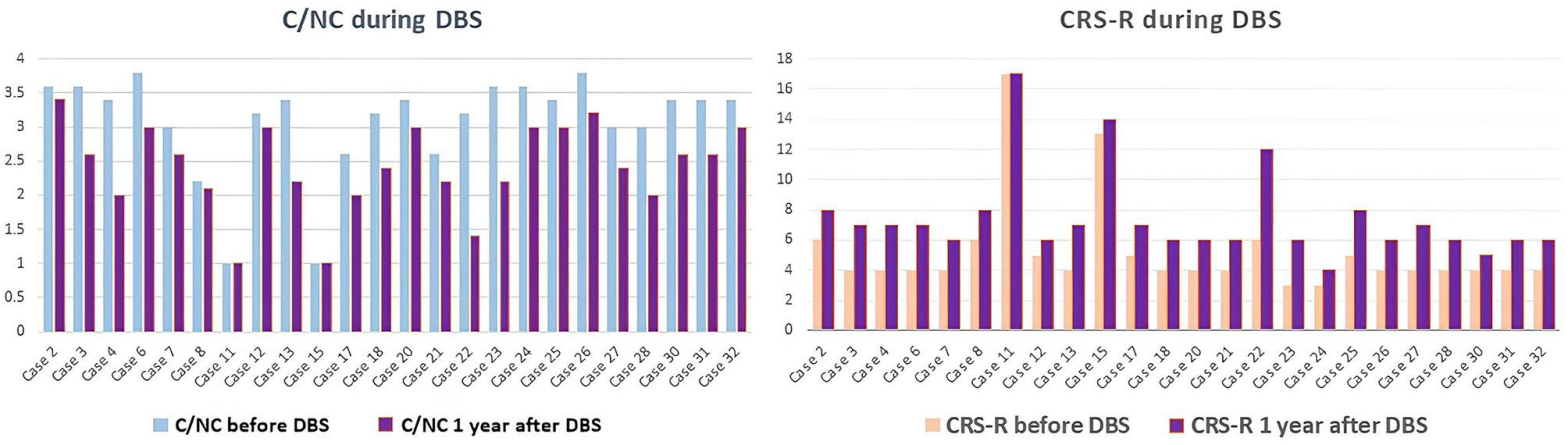
C/NC and CSR-R scores preoperatively and one year after CM-pf DBS in non-responders, showing some improvement regarding consciousness level.

All 32 stimulated patients showed some improvement one year after the DBS implantation, even non-responders (Fig. 4). Using C/NC scale, improvement was significant in the following subcategories: pain, tactile, visual, and auditory (*p* = *0.05*). Regarding other subcategories, command responsivity was observed only when the consciousness level was closer to awareness. Using the CRS-R scale, improvement was significant in the following subcategories: motor, auditory, and visual (*p* = *0.05*). Like previously, subcategory communication was observed only when the consciousness level in patients was improving closer to the level of awareness (Table 4). Furthermore, we compared the group of patients who underwent CM-pf DBS and patients who did not fulfil inclusion criteria. No significant difference was observed when comparing the initial C/NC and CRS-R score of both groups. All patients who underwent CM-pf DBS had some improvement according to C/NC and CRS-R scales (Table 5), as was mentioned before, while patients who did not fulfil inclusion criteria and thus weren’t implanted during follow-up showed no improvement according to C/NC and CRS-R scale.

In our cohort, the DBS system was extracted three years after the implantation, regardless of the result. Patients showing no improvement in their consciousness level are regarded as non-responders. As the non-responders show no improvement between second and third-year follow-up, we consider the DBS system to be redundant. In addition, their DBS systems were explanted after three years to avoid potential complications (skin erosions and consequential infection in these mostly cachectic patients). Regarding responders, after reaching the level of full awareness, the DBS system is found to be redundant as there is no need to stimulate the patient anymore.

Regarding complication, five of the UWS patients died after DBS implantation; one due to pneumonia four months postimplant, one due to sepsis 18 months postimplant, one after a cerebrovascular insult 33 months postimplant, one due to pneumonia 12 months postimplant, and one at eight months post-implant due to heart failure. Two UWS patients and one MCS patient had seizures during DBS, which were controlled with antiepileptic drugs, allowing DBS to proceed. Four patients suffered from hypoxic myoclonic jerks before DBS; myoclonus symptoms diminished and finally disappeared several days after the initiation of the CM-pf DBS. In one patient, who recovered to the level of awareness, an intrathecal Baclofen pump was implanted due to worsening spasms. There was no intra- or postoperative intracranial bleeding or postoperative infection, such as meningitis or wound infection.

## Discussion

In this study, we present our ten years of experience in treating DoC patients using CM-pf DBS. Keeping in mind the ethical criteria and seriousness of DoC, our previous results, as well as results of other groups worldwide, showed encouraging success. Several points are important to raise concerning the arousal response, the CM-pf as a target, stimulation parameters, mainly frequency, assessment scales, and timing of DBS.

In the cohort of initially 36 DoC patients, who fulfilled inclusive criteria, 31 were in UWS, and five were in MCS. In the group of patient who did not fulfil inclusion criteria (47 patients), there was no patient in MCS. The majority of included patients, whether candidates for DBS or not, suffered DoC due to ischemic brain lesions. Furthermore, the frequency of the DoC patients is higher in patients who suffered from ischemic lesion due to the observation that traumatic brain injury patients have much better chance of recovery of consciousness^3–5^.

Seven out of 32 patients had substantial improvement in their consciousness level. The results of the Japanese authors were eight patients out of 21 stimulated that responded to a verbal command, which is more than in our study, however their patients were all in a UWS after traumatic brain injury^22^. On the other hand, most patients in our study suffered DoC due to CA. The reason of low rate of patients who really improved could be in a long period from injury to DBS (13.16 ± 24.41 months, range 2–137 months), as well as the fact that CRS-R score overall was quite low and in a discrepancy of the patients level of consciousness between 14 patients stimulated from 2011 to 2017, described in our previous paper and 18 patients which we stimulate as a continuation of cohort in period from 2018 to 2022.

In a second part of cohort, we had only one patient in MCS and the and the level of consciousness according to RDR and CRS-R scale was much lower. The tendency could be that priority for stimulation have patients with better level of consciousness and much earlier after incident. We emphasize and encourage early treatment using CM-pf DBS for DoC patients, especially in MCS patients, with higher level of consciousness, and with shorter period in-between incident and DBS procedure.

All DBS-treated patients had statistically significant improvement in a CRS-R, in auditory and visual subcategories, and in the C/NC scale in pain, tactile, visual, and auditory. However, these improvements were of minimal clinical importance. Minimal clinical importance refers to a lack of consciousness recovery what is the most important issue for the patients, caregivers and therapists, meaning statistically significant improvement in separate subcategories of clinical scores doesn’t necessarily lead to consciousness improvement. The group of 47 patients who did not meet the criteria according to neurophysiological testing during the follow-up did not present any improvement in their state of consciousness. Therefore, neurophysiological tests can be used not only as a part of the inclusion criteria for DBS but also as a relatively reliable way of making the prognosis of patients with DoC. The neurophysiological testing could be used to help objectively distinguish the difference between MCS and UWS because the absence of evoked potentials is not compatible with MCS. One of the critical questions of the study we did not answer is why the level of consciousness was improved only in seven out of 32 patients (approximately 21%) who underwent DBS. All 32 patients fulfilled the entry criteria. We must admit that our entry criteria were a good way to indirectly test the functional integrity of the cortex and brainstem, but we did not have a specific marker to test essential elements predicting the reversibility of DoC.

### Arousal response

Electrical stimulation was programmed until we induced an arousal response in each patient, which helped us to simulate the target with a sufficiently strong current. As consciousness gradually improved, we observed that arousal reaction could not be elicited as it was before, using the previously mentioned variety of stimulation parameters. With further improvement from MCS to full awareness, the arousal effect completely disappeared when turning the stimulation on. Interestingly, the fully aware patients reported no sensations when turning the stimulation on. Additionally, in the group of responders, no consistent behavioral or affect change was observed that could be linked to the stimulation paradigm. Additionally, the arousal response was described in the literature not only during stimulation of thalamic nuclei but also while stimulating some other neuroanatomical structures, like rostral thalamic nuclei, mesencephalic reticular formation, putamen, and globus pallidus^15,17,21–23^. Furthermore, arousal response could be induced not only in patients with DoC, but also in patients under general anesthesia^33^. Therefore, it seems that arousal response depends not only on the stimulated neuroanatomical structure but also on a consciousness level.

### Frequency of stimulation

Low-frequency stimulation was used, 25–40 Hz, as described in a paper from Tsubokawa and Yamamoto^20–23^. Counting the number of patients with DoC in the literature treated with DBS, low-frequency stimulation was more commonly used than high-frequency stimulation, for example Hassler 50 Hz in 2 UWS patients^15^, Tsubokawa and Yamamoto 25–40 Hz in 21 and 8 UWS patients^20–23^, Cohadon 50 Hz in 25 UWS patients^18^, Magrassi < 40 Hz in 2 UWS and 1 MCS patients ^26^, Chudy 25 Hz in 4 MCS and 10 UWS patients^27,28^, Lemaire 2018 30 Hz in 4 MCS and 1 UWS^34^. Arnts and al. showed in one MCS patient that low-frequency stimulation of 50 versus 100 Hz stimulation induces a pattern in magnetic encephalogram that resembles more as the healthy control^29^. Still, it was observed in nine MCS patients that DBS with 100 Hz improves the functional connectivity of EEG but did not mention low-frequency usage^35^. On the other hand, daytime use of continuous high-frequency stimulation of 130 Hz in an MCS patient was shown to be behaviorally and electrophysiological effective^25^. Therefore, it looks like the benefit of using low or high frequency remains unclear.

### CM-pf nuclei as a target for stimulation

Despite numerous studies and hypotheses, the phenomenon and neurobiology of consciousness are not fully understood. The role of intralaminar thalamic nuclei in arousal during wakeful states has been exceptionally well described in rodents^36^. Namely, it was suggested that the CM-pf complex, the main part of the intralaminar nuclei, provides particularly strong connections with cortical and subcortical structures^37^. Injury of the thalamus intralaminar nuclei can result in various deficits ranging from cognitive disturbances to DoC, depending on the extent of the injury^38^. Therefore, it has been speculated that an incomplete loss of thalamic neurons after brain injury may damage thalamocortical and thalamostriatal activity^39^, leading to a negative feedback loop resulting in a loss of disinhibition of the thalamus from pallidum^40^. Therefore, we believe that the selection CM-pf complex of the thalamus seems justified because of its effect on the widespread thalamocortical and thalamostriatal circuitry, and additional modulatory systems^41^. Consequently, such intervention could reverse aberrant signals cascade and facilitate the restoration of arousal regulation^40^.

We used CM-pf for stimulation, as Tsubokawa and Yamamoto described in their papers^20–23^. The stimulation parameters imply that the electric field around the stimulating electrode covers a broad area of the central thalamus. Yamamoto explained that the angle between the AC-PC plane and the lead trajectory is sharp, so most of the active contacts were within the thalamus covering from central to rostral part of nonspecific intralaminar nuclei (Yamamoto, personal communication). Tasserie and al. used DBS of the central thalamus in anesthetized nonhuman primates and induced arousal response^42^. They found that a higher stimulation intensity enhanced clinical arousal score, while stimulation of the thalamus's ventrolateral nucleus did not affect arousal score. Redinbaugh et al. found that in macaques, central lateral thalamic nuclei stimulation arouses from anesthesia and presents the correlation of thalamic and deep-layer cortical spiking, which could be valuable to put in consideration of CL nuclei for simulation in patients with DoC^43^. Furthermore, important differential effects on behavioral arousal and cortico-cortical physiology when directly comparing 50 and 200 Hz stimulation in the same nuclei were demonstrated^43^. Additionally, several studies confirmed their main results in anesthetized non-human primates^42,44^. Furthermore, a study in awake and behaving non-human primates demonstrated differential effects on performance when stimulating within CL versus CM, based on biophysical modeling of the target structures^45^. There was a good example of how the results from studies on macaques directed the discoveries for use of DBS in clinical practice such as, subthalamic DBS for patients with Parkinson disease^46,47^. However many discoveries in stereotactic neurosurgery were found out by serendipity, mainly because of human specific neurological and psychiatric diseases^48^.

Moreover, the DBS of central thalamus restored a broad dynamic repertoire of spontaneous resting-state activity, described as a signature of consciousness^42^. In recent literature about DBS in patients with DoC, authors used more bilateral CM thalamic nuclei stimulation^29^. We implanted the electrode in only one hemisphere, left in right-handed patients, but in patients who had significant damage after brain trauma, predominantly in one hemisphere, we placed the electrode in the more preserved one.

### Assessment scales

For the follow-up, we use CRC-R because this scale covers even slight changes in the level of consciousness, and it is worldwide accepted, so it allows our data to be comparable with data from other centers. However, the CRS-R scale is not easy to use and is time-consuming, so educating medical staff and caregivers who use the CRC-R scale is mandatory. Therefore, the organization of patient evaluation in other institutions could become very difficult and, in some instances, almost impossible.

### Time of beginning DBS after the incident and ethical dilemmas

One of the crucial questions is when is the right time is to start DBS in patients with DoC. After the various type of brain lesions, either traumatic or ischemic, several underlying processes of brain tissue repair (apoptosis, microstructural glial reactivity, changes in the extracellular matrix, etc.), plasticity (sprouting, myelination, dendritic plasticity), and reorganization occur^49^. Still, delaying DBS in patients with DoC increases the probability of developing irreversible joint and muscle changes leading to severe disability, even when patients reach the level of consciousness. So, the patients become conscious of their severe disability developing self-awareness syndrome, described in the literature as one of the main ethical problems^50^. Therefore, it is less likely that the development of self-awareness syndrome will appear if we start with DBS a relatively short time after the incident.

However, such early use of DBS in some patients with DoC could hide the possibility of spontaneous recovery of consciousness. While indeed the best results in two CA MCS patients were obtained within three months from injury, and a month after the stimulation, an additional three CA UWS patients in which DBS was implanted from 4 to 24 months after initial injury reached the level of awareness in between 6 and 12th month of stimulation, meaning the consciousness recovery of 3 post-ischemic lesions patients started from 10 to 36th months. In addition, in both TBI patients DBS was implanted 11 and 12 months after injury, and those patients also reached the level of awareness between the 6th and 12th month of stimulation, meaning the consciousness recovery started approximately 17 months after initial injury, which differentiates from previously described spontaneous recovery, occurring within a year after the initial injury. The Japanese group reported success in 8 VS patients from a group of 21 stimulated patients, improving the awareness; they began DBS 4–8 months after injury. Furthermore, in 87 patients who fulfilled neurophysiological criteria and were not stimulated, spontaneous recovery was not recorded^22^. Schiff et al. and Magrassi et al. started DBS from 6 months to 6 years after injury but without substantial improvement in patient awareness^25,26,34^.

Is it ethical that the patient becomes aware of their severe disabilities? We claim that this is not a specific ethical question for patients with DoC. We try in everyday neurosurgical practice to save the life of severely brain traumatically injured patients, although we know that some will survive with very severe neurological deficits. Our group emphasizes the need for and importance of early intervention due to irreparable changes in the brain and musculoskeletal system, in order to avoid bed-ridden complications like pneumonia, decubitus, etc. In addition, our previously published results, combined with a review of literature, suggest that implementation of DBS in UWS or MCS patients a long time after injury offers very low possibility for the recovery of consciousness to the level of responsiveness, and certainly does not prevent irreversible changes of the musculoskeletal system.

Furthermore, DBS is not the first line of treatment for patients with DoC, neither for patients after cardiac arrests nor after brain trauma. Therefore, we implemented the same rule as for any other functional neurosurgical procedure, that is, to first exhaust all non- or less-invasive therapies for DoC patients. This includes the administration of amantadine, the evidence-based therapy for patients with DoC^51^, or intrathecal delivery of baclofen^52,53^, where spasms were the main reason for patients' inability to perform aoluntary movement**.** When all non-invasive or less invasive procedures have been exhausted, we consider whether the patient is a candidate for DBS.

This also raises the question of who has the legal authority to decide on an invasive procedure such as DBS in a patient with a DoC. An honest discussion with caregivers, mainly members of the family, about the invasiveness of procedures like DBS and the experimental state of the study, as well as the possibility that the patient reaches the level of awareness spontaneously, is crucial for the correct and honest relationship between clinicians and the patient´s caregivers.

Several limitations of the presented study should be mentioned, such as the heterogeneity of patients concerning etiology, different ages at the time of the initial injury, and different times from initial injury to DBS implantation. Indeed, we are aware of the main limitation of our study, regarding spontaneous recovery vs DBS, and we do not make a firm and final conclusion about DBS in patients with DOC but note that further research is needed.

## Conclusion

Our results showed some potential for DBS in patients with DoC as a therapy. We have as yet no definitive answer to many questions regarding DBS in a patient with a disorder of consciousness, such as; the exact definition of which neuroanatomical structure must be stimulated, what are optimal parameters of stimulation, when to start with DBS, and how to choose more refine the inclusion criteria.

To find the answers to those questions and solve the many issues with such therapy, cooperation between worldwide medical centers interested in the possibilities of DBS in patients with DoC should be mandatory.

Future research in larger, more homogeneous groups of patients is necessary to determine the risk–benefit ratio for performing DBS in individual patients with severe brain injury.

## Data Availability

The data generated for this study are available on request to the corresponding author.
